# HIV Vaccination: A Roadmap among Advancements and Concerns

**DOI:** 10.3390/ijms19041241

**Published:** 2018-04-19

**Authors:** Maria Trovato, Luciana D’Apice, Antonella Prisco, Piergiuseppe De Berardinis

**Affiliations:** 1 INSERM u1016, Institut Cochin, 27 Rue du Faubourg Saint Jacques, 75014 Paris, France; 2 Institute of Protein Biochemistry, C.N.R., Via Pietro Castellino 111, 80131 Naples, Italy; l.dapice@ibp.cnr.it (L.D.); p.deberardinis@ibp.cnr.it (P.D.B.); 3 Institute of Genetics and Biophysics A. Buzzati-Traverso, C.N.R., Via Pietro Castellino 111, 80131 Naples, Italy; antonella.prisco@igb.cnr.it

**Keywords:** HIV-1, vaccines, clinical trials, immune correlates

## Abstract

Since the identification of the Human Immunodeficiency Virus type 1 (HIV-1) as the etiologic agent of AIDS (Acquired Immunodeficiency Syndrome), many efforts have been made to stop the AIDS pandemic. A major success of medical research has been the development of the highly active antiretroviral therapy and its availability to an increasing number of people worldwide, with a considerable effect on survival. However, a safe and effective vaccine able to prevent and eradicate the HIV pandemic is still lacking. Clinical trials and preclinical proof-of-concept studies in nonhuman primate (NHP) models have provided insights into potential correlates of protection against the HIV-1 infection, which include broadly neutralizing antibodies (bnAbs), non-neutralizing antibodies targeting the variable loops 1 and 2 (V1V2) regions of the HIV-1 envelope (Env), polyfunctional antibody, and Env-specific T-cell responses. In this review, we provide a brief overview of different HIV-1 vaccine approaches and discuss the current understanding of the cellular and humoral correlates of HIV-1 immunity.

## 1. Introduction

Since the first published report [1] (1981) of Acquired Immunodeficiency Syndrome (AIDS) in five homosexual men in Los Angeles being treated for *Pneumocystis carinii* pneumonia, Human Immunodeficiency Virus type 1 (HIV-1) has given rise to one of the most deadly infectious disease in the human history, causing about 35 million deaths. The latest estimate, available on the UNAIDS website (available online: http://www.unaids.org/en), tells us that over 36 million people worldwide were living with HIV/AIDS at the end of 2016 and 1 million persons died of AIDS in 2016, with sub-Saharan Africa remaining the area most severely affected by the HIV/AIDS pandemic. Currently, increasing numbers of HIV-infected individuals have access to the life-saving, highly effective antiretroviral therapy (HAART) that can suppress the plasma viremia and reduce the risk of AIDS and transmission of HIV [2]. However, the persistence of HIV-latent reservoirs makes the complete eradication of the virus in infected individuals receiving HAART extremely problematic [3,4].

**Figure 1:**
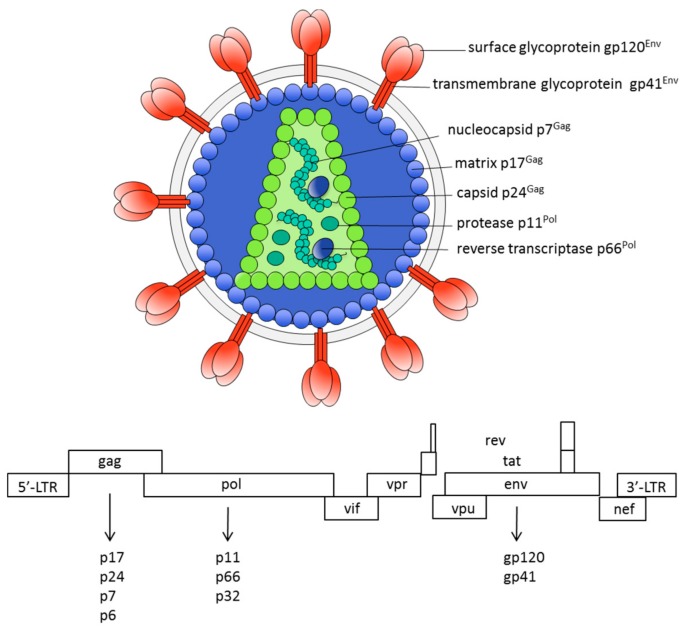
Human immunodeficiency virus type 1 (HIV-1) genome and virion.

HIV-1 is an enveloped RNA virus, a lentivirus belonging to the Retroviridae family. The virus was isolated and subsequently identified as the etiologic agent of AIDS in 1983 [5]. The viral genome, consisting of two copies of a single-stranded RNA, codes for structural (Gag, Pol, and Env), regulatory (Tat and Rev), and accessory proteins (Vpu, Vpr, Vif, and Nef) (Figure 1). As in other enveloped viruses, binding of the virus to cell-surface receptors is mediated by the envelope glycoproteins, which play a critical role in initiating the viral infection [6]. HIV-1 primarily infects CD4+ T-cells, macrophages, and dendritic cells, causing functional defects and damage to the immune system.

A safe and effective vaccine would be an invaluable tool to stop the HIV/AIDS pandemic. So far, only one HIV-1 vaccine clinical trial—the Thai Phase III RV144—demonstrated some efficacy of vaccination against HIV-1 acquisition [7]. Currently, the analyses of vaccine-induced immune correlates of protection in humans and non-human primate (NHP) models are guiding different HIV-1 vaccine approaches [8,9,10]. In this review, we provide a general overview of the recent advances in HIV-1 vaccine development, with a focus on the immunological mechanisms.

## 2. The HIV Vaccine Problem: Roadblocks and Main Challenges

The development of an HIV-1 vaccine is a daunting challenge that faces serious roadblocks. Traditional approaches to viral vaccine development are unworkable or inefficacious. Safety concerns rule out the use of the HIV-1 in live/attenuated vaccine formulations. The high propensity of the HIV-1 to mutate to escape immune response results in a high degree of genetic diversity that demands broadly cross-reacting neutralizing antibodies to prevent infection and, to date, such broadly neutralizing responses proved arduous to achieve by vaccination. The early establishment and persistence of latent virus reservoirs during infection is a potent mechanism of viral immune escape as well as the structure of the envelope (Env) protein. The highly glycosylated structure of the trimeric Env and the presence of Env immunodominant variable regions shield broadly neutralizing antibody (bnAb) epitopes and drive the immune response away from conserved regions [11,12].

## 3. The Working Hypotheses on How to Protect from HIV/AIDS

Virus-specific nAbs represent the key immune correlates of protection for most successful viral vaccines. HIV-1 bnAbs have been described that target different regions of the Env trimer, namely, the CD4 binding site, the variable loop 1 and 2 (V1V2) glycans and the V3 glycan on the gp120 protein, and the gp120–gp41 interface [13]. Formulations able to induce bnAbs capable of neutralizing the majority of HIV strains are a main aim of vaccine development efforts [14].

Along with protective antibodies, also the anti-HIV CD8 T-cell response is relevant for the control of HIV replication, although the specific mechanisms contributing to this suppressive activity are yet to be fully elucidated (recently reviewed by McBrien et al. [15]). The role of CD8 T-cells has been emphasized by preclinical studies [16,17,18,19], and by the data collected during HIV infection reporting that a decline of viremia occurs after the induction of virus-specific CD8 T-cells, thus suggesting that CD8 T-cells are involved in the initial control of infection [20]. Moreover, clinical trials have provided insights into potential correlates of protection against the HIV-1 infection, including polyfunctional immune responses [10].

Thus, an ideal vaccine should contain immunodominant epitopes conserved among the different clades and be able to elicit both bnAbs and a broad CD8 T-cell response.

## 4. Exploiting Germinal Center (GC) Responses for Eliciting bnAbs

bnAbs can prevent infection and, therefore, are of great importance for therapeutic strategies against HIV. It is known that between 10% and 50% of individuals develop nAbs, but only a small percentage of HIV-infected individuals develop protective nAbs 2–3 years after infection [21]. The sole target of nAbs is the Env complex—a heterotrimer composed of three molecules of gp120 and three molecules of gp41—which is responsible for mediating viral entry into host cells [14].

The importance and the role of nAbs have prompted studies aimed at their identification. Two technological approaches were instrumental in the discovery of new nAbs: (1) sera-screening programs on large cohorts of infected individuals and (2) methods to culture and activate individual memory B-cells combined with sorting Env-specific B-cells and subsequent screening of culture supernatants for neutralizing activities [22,23,24]. These approaches allowed the identification of a new generation of highly potent and/or broadly cross-reactive human monoclonal antibodies. Moreover, the advancements in B-cell cloning demonstrated that broad and potent nAbs generally comprise less than 1% of the HIV (Env)-specific memory B-cell repertoire [25].

Overall, bnAbs have peculiar characteristics. They generally have long heavy-chain complementary-determining region 3 (HCDR3) generated at the stage of variable-diversity-joining recombination. Given the low frequency of long HCDR3 in naive B cells, the precursors to many bnAbs constitute a rare population in the naive repertoire [26]. Moreover, bnAbs are highly somatically mutated. Whereas most human antibodies may carry 15–20 heavy-chain-variable gene somatic mutations after undergoing affinity maturation, bnAbs carry 40–100 VH gene mutations, which are essential because reversion to their germline sequence reduce neutralizing efficacy and breadth [27,28,29]. Most mutations occur in the framework regions, where mutations are usually poorly tolerated because of the risk of destruction of the structure of the variable domain [30]. Recently, it has been reported that antibodies recognizing the V2 region at the apex of the HIV Env trimer are among the most common bnAbs specificities during chronic infection, and many exhibit remarkable breadth and potency [31].

In infected individuals, nAbs directed against the transmitted-founder virus rapidly select virus escape mutants, which in turn induce new antibody specificity. After years of infection, the coevolution of the virus and the antibody response results in bnAbs [13]. This process is difficult to recapitulate in a vaccination protocol. The unusual features of bnAbs—including high levels of somatic hypermutations (SHM) that suggest prolonged maturation pathways, a long HCDR3, and/or polyreactivity and autoreactivity, whose acquisition correlates with the evolution of antibody neutralization breadth [32,33,34]—are typically associated with autoantibodies. Thus, the development of bnAbs might be constrained by host immune tolerance checkpoints [32,35].

The majority of B-cells expressing self-reactive B-cell receptor (BCR) are eliminated by central and peripheral mechanisms of immune tolerance in order to limit autoantibody production, including those that take place within the germinal center (GC) for the antibody affinity process [36]. Within GCs, antigen-activated B-cells undergo active SHM, diversifying the immunoglobulin genes and generating somatically mutated high-affinity antibody-secreting plasma cells and memory B-cells. Extensive SHM is required to generate HIV-1 bnAbs [30], and antibody affinity maturation is dependent on the interaction of GC B-cells with the cognate GC T follicular helper (Tfh) cells [37,38]. Therefore, the generation of bnAbs might be under the control of Tfh cells. Of note, preclinical and clinical studies have provided evidence that the frequency of GC B-cells and Tfh cells correlated with the generation of bnAbs during HIV and simian immunodeficiency virus (SIV)/simian-human immunodeficiency virus (SHIV) infection [39,40,41].

Locci and colleagues identified, in normal individuals, a subset of blood-circulating memory Tfh cells that are highly functional for B-cell help and correlated with the ability to generate HIV-1 bnAbs in HIV+ individuals [40], suggesting that vaccine candidates might target this population to provide maximal B-cell help, drive high levels of SHM, and thus induce bnAbs. Cohen and collaborators [42] have shown that early preservation of a specific subset of peripheral Tfh cells strongly correlated with the breadth of nAb responses during chronic HIV-1 infection, suggesting that maintenance of this subset would be necessary to preserve the early B-cell activation profile essential for the affinity maturation, SHM, and generation of bnAbs. In addition, plasma levels of CXCL13 chemokine were associated with plasma bnAbs detected during the chronic phase of HIV-1 infection [42]. CXCL13 has been identified as a plasma biomarker of GC activity, being associated with GC frequencies in humans and animal models [43]. GC Tfh cells produce CXCL13, and the chemokine levels correlate with the development of HIV bnAbs [43]. Moody et al. also found that HIV-infected individuals with plasma bnAbs had higher frequencies of resting memory Tfh cells compared to HIV+ individuals with low or no bnAbs [44].

These findings strengthen the concept of eliciting better GC responses through new vaccines aimed at inducing HIV-1 bnAbs [45].

## 5. The “Omic Approach”

The improvement in nucleic acid sequencing technologies has given an invaluable contribution to collect the complete sequence of HIV-1 isolates from patients throughout the world. An overview of the diversity of HIV genome has been reported by Tongo and Burgers [46].

The search for common epitopes for vaccine design represents a bioinformatic challenge, which promoted the development of ad hoc tools to cope with the pathogen’s genome diversity [47]. Viral genome sequencing provided hints about drug-resistant strains useful to predict response to drug treatments [48] and revealed mechanisms of viral adaptation to the host response [49]. Proteomic analyses integrate the genomic information with data regarding post-translational modifications, temporal expression, and amount of expression of viral protein [50]. The interaction between virus and host cell has been captured in the “interactome” [51], a mass of data collected from HIV-infected lymphocytes. From these “omics” data, it is possible to retrieve information about the effect of the viral infection on the host cell’s gene modifications and protein expression, with the aim to design new candidate vaccines, and to block the viral replication.

Preclinical research using bioinformatics-derived candidate vaccines have been reported by Azizi et al. [52]: starting from the hypervariable sequences of Env and Gag HIV proteins (identified by comparison of more than 200 virus genomic sequences), 15–27 amino acid peptides have been selected. Tests in NHPs demonstrated the induction of a broad cellular immune response and the production of nAbs with moderate performance [52]. A similar response was observed by Rosario et al. [53] using long synthetic peptides corresponding to conserved regions of HIV proteins.

The first HIV vaccination clinical trials attempted to induce nAbs by priming and boosting with recombinant gp120 from different clades. However, neutralization activity was obtained only against a small subset of viral strains, while patient-derived isolates were not neutralized [54].

The elusive goal of inducting bnAbs has been the subject of varied and intense research efforts. In the early 1990s, nAbs were identified using phage display libraries and human hybridomas, whereas the second-generation nAbs have been obtained by screening a large cohort of infected individuals [25]. Memory B-cells were then screened for neutralizing activity and genes encoding the antibodies of interest were cloned [55]. The X-ray structure resolution of VRC01 nAb and the deep sequencing of antigen specific B-cell immunoglobulin light and heavy chains, the so-called antibodiome, helped to define the nAb binding sites and suggested that nAbs follow a common pathway of maturation [56]. The analysis of nAbs revealed that glycosylation is important in epitope binding, and neutralization ability is increased by deglycosylation [57].

Overall, the huge mass of omic data fostered the production of bioinformatic tools to predict the best candidate epitopes for vaccine design [58,59].

## 6. Learning from the Clinical Trials

To date, only six HIV-1 vaccine candidates have been tested in efficacy trials, with no licensed vaccine yet [60] (see Table 1).

**Figure 2:**
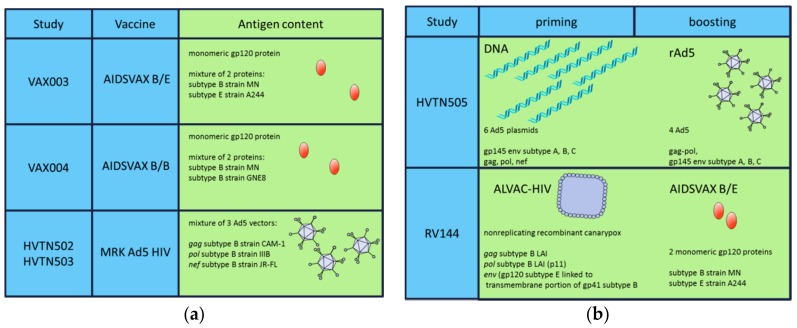
Vaccine regimens tested in HIV-1 clinical trials. (**a**) Antigen compositions; (**b**) Priming/boosting strategies.

The first preventative HIV vaccine candidates tested in efficacy trials consisted of bivalent recombinant Env gp120 in alum: AIDSVAX subtype B/B in the VAX004 clinical study and AIDSVAX subtype B/E in the VAX003 trial (Figure 2a). VAX004 enrolled ~5400 men who have sex with men (MSM) and high-risk women in the United States and the Netherlands [61,62]. Concurrently, the VAX003 study tested AIDSVAX B/E vaccine in ~2500 injection drug users in Thailand [63,64]. VAX004 and VAX003 aimed at inducing HIV nAbs, based on the hypothesis that nAbs would prevent HIV-1 acquisition. Even though high levels of binding and nAbs (in VAX003 and VAX004) and antibody-dependent cell-mediated virus inhibition (in VAX004) were observed, both candidates failed to show efficacy in preventing HIV acquisition due to their narrow specificity (reviewed in [65,66]).

The disappointing results of the vaccines consisting of recombinant Env shifted the focus of research on CD8 T-cell immune responses, with the aim to control viral replication. The Step study (HVTN 502) and the Phambili trial (HVTN 503) tested replication-defective recombinant adenovirus type 5 (Ad5) vector-based vaccines: the MRKAd5 HIV-1 gag/pol/nef clade B vaccine (Figure 2a). The Step study enrolled ~3000 MSM and high-risk women in USA, while the Phambili trial enrolled ~800 heterosexual men and women in South Africa [67,68]. Both trials aimed primarily to reduce the post-infection viral load but were early interrupted as an increased infection risk was observed in vaccinees, being higher in vaccinated men who were uncircumcised and with preexisting immunity to Ad5 (reviewed in [65,66]).

To overcome the narrow specificity of the antibody response observed in VAX004 and VAX003 studies and with the aim to stimulate both arms of the immune response, the HIV Vaccine Trials Network 505 (HVTN 505) tested a heterologous prime/boost approach (Figure 2b). A DNA vaccine expressing clade B gag/pol/nef, and env from clades A, B, and C, was used to prime multiclade *r*Ad5 HIV vaccines. The HVTN 505 trial enrolled ~2500 Ad5 seronegative (Ad5-nAbs titers < 1:18) and circumcised MSM and transgender women [69]. No increased risk of infection was observed, however, the vaccine regimen did not show efficacy (reviewed in [65,66]).

The first, and so far only, clinical trial that demonstrated a protective effect on HIV-1 acquisition was the RV144 trial [7], a prime-boost regimen of a recombinant canarypox vector (ALVAC-HIV) and the AIDSVAX B/E Env gp120 protein in alum (Figure 2b). The RV144 trial, which enrolled ~16,000 high-risk male and female volunteers in Thailand, demonstrated 31.2% vaccine efficacy at 42 months against HIV acquisition in Thai men.

Vaccine efficacy was associated with non-nAbs to V1V2 regions of HIV-1 Env, Fc-mediated effector responses, high levels of V2-specific antibodies mediating ADCC (antibody-dependent cellular cytotoxicity), HIV-1 V2-specific IgG3 antibodies, and Env-specific CD4+ T-cell responses, while Env IgA antibodies were seen to directly correlate with HIV-1 infection [8,70]. It is possible that non-nAbs targeting the V1V2 regions neutralize only a narrow subset of viruses given the aa variability in this region.

The lessons from RV144 suggested that the HIV-1 V2 region could be a target site to potentially induce antibodies able to block HIV-1 acquisition.

## 7. Current HIV-1 Vaccine Approaches

The modest efficacy of the RV144 HIV-1 vaccine candidate and the analyses of vaccine-induced immune correlates of protection in humans and NHP models [8,9,10] are currently guiding the development of novel HIV-1 vaccines [71]. Preventative and therapeutic strategies have been pursued and investigated into test-of-concept clinical trials with the aim to provide protection against HIV-1 [72,73]. RV144 regimen, novel vaccine regimens based on Ad26 viral vector, mosaic immunogens, and gp140 protein, HIV-1 SOSIP trimers, and passive administration of monoclonal antibodies are amongst the latest approaches to HIV-1 prevention and treatment. Here, we report some HIV immunotherapeutic current approaches (listed in Table 2).

### 7.1. RV144 Follow-On Approaches

Based on the hypothesis that non-nAbs targeting the V2 region could block HIV-1 acquisition, the RV144 follow-on clinical trials RV305 and the HVTN studies HVTN097, HVTN100, HVTN702, and HVTN107 were designed to elicit enhanced V2-specific antibody responses matched with the local viral strains, using the RV144 vaccine regimen.

The RV305 enrolled HIV-1 uninfected RV144 vaccinees to test the efficacy of RV144 vaccine boosting on B-cell responses. Boosts with an Env immunogen that binds the unmutated common ancestor (UCA) of bnAbs resulted in the expansion of memory B-cells specific for the CD4 binding site of Env, with some degree of tier 2 neutralizing ability to circulating HIV (so-called tier 2 viruses), suggesting that the RV144 regimen primed long-lived memory B-cells that could be recalled with repetitive boosting [74].

To evaluate the safety and immunogenicity of the RV144 regimen in other regions, the HVTN097 study enrolled HIV-1 uninfected adults in South Africa. Env-specific CD4+ T-cell responses were induced in South Africans comparable, if not better, to those induced by RV144 in Thais [75].

HVTN100 phase 1/2 clinical study aimed to provide enhanced and sustained protection against the predominant HIV subtype C in HIV-1 uninfected volunteers in South Africa. HVTN100 tested the combination of ALVAC-HIV (vCP2438)/bivalent subtype C with MF59-adjuvanted gp120 protein and included a 12-month boost [76]. Based on promising results, a phase 2b/3 trial (HVTN702) testing HVTN100 efficacy in HIV-uninfected South Africans is ongoing, while the HVTN107 trial is testing the HVTN100 regimen with different adjuvants.

### 7.2. Mosaic HIV-1 Vaccines

Polyvalent HIV-1 Gag, Pol, and Env mosaic immunogens expressed by replication-incompetent adenovirus serotype 26 (Ad26) vector and optimized to provide maximal coverage of potential T-cell epitopes (PTEs) represent a promising strategy to increase the breadth and depth of epitope-specific cellular immune responses against a pathogen with a high degree of genetic diversity such as HIV-1 [77]. Binding, neutralizing, and non-neutralizing vaccine-elicited antibodies all correlated with protection in NHPs [78], suggesting that multifunctional antibodies might contribute to give protection against different HIV-1 circulating strains.

Based on this concept, novel vaccine strategies are entering human clinical trials, including priming with replication-incompetent Ad26 adenoviral vector vaccines or modified vaccinia Ankara (MVA) vectors expressing mosaic immunogens and boosting with Env gp140 protein, with the goal to provide protection against different circulating viral isolates by inducing binding, neutralizing, and functional non-nAbs and different cellular immune responses [73]. These strategies might be useful to overcome the narrow specificity and to evade the preexisting immunity observed in *r*Ad5-based HIV-1 vaccine candidates.

### 7.3. SOSIP Trimers as Platform to Induce bnAbs

Among the recent immunization strategies to induce bnAbs, biochemically stabilized soluble native-like HIV-1 Env trimers are under investigation, including the SOSIP.664 gp140 trimer [79]. Recombinant soluble native-like trimers closely resembling the native HIV-1 Env spikes provide details on how bnAbs recognize these epitopes on the viral Env surface, and thus represent a powerful platform to guide the design and the development of new structure-based immunogens. In the SOSIP design, gp120 and gp41-ectodomain subunits are covalently linked through an intermolecular disulfide bond named SOS and stabilized in the prefusion state by an amino acidic mutation (named IP) to avoid the display of immunodominant non-nAb epitopes, resulting in a native trimeric conformation recognized by bnAbs [80]. Using this technique, SOSIP.644 Env trimers based on neutralization-resistant circulating Tier 2 subtype A (BG505) and B (B41) viruses were engineered and have been shown to induce autologous nAbs in rabbits and macaques [81,82], with the autologous response against BG505 mapping into a glycan hole region [82]. Autologous Tier 2 nAbs were also induced in NHPs immunized with a modified BG505 SOSIP designed to improve the stability of the protein, the BG505 SOSIP.v5.2 [39].

These findings proved that SOSIP trimers might be a useful strategy to be pursued for the design of structure-based immunogens aimed at eliciting broadly active nAbs. New strategies to broaden the nAb response are under investigation, including simultaneous or sequential administration of combined SOSIP trimers based on different clades, Env lineage-based vaccines selected from patients developing bnAbs, and HIV immunogens targeting specific germline BCRs [83]. In addition, these strategies would benefit from structure-based computational design exploiting molecular modeling programs, as described by Kulp et al., with the goal to minimize the conformational dynamics that would expose non-neutralizing epitopes and retaining the structural integrity of native-like trimers [84].

### 7.4. Passive Immunotherapy and Pre-Exposure Prophylaxis (PrEP)

So far, the development of prophylactic vaccines able to elicit bnAbs remains a challenge. In this context, it has been proposed that passive antibody prophylaxis, an old therapeutic strategy, may represent a promising alternative to vaccination. BnAbs showed potent prophylactic efficacy in animal models. Multiple studies in NHP and mouse models have shown that bnAbs passively administered in several ways (via intravenous, vaginal, rectal, and oral routes) provide protection against HIV challenge [85,86,87,88]. A large ongoing study named “Antibody-mediated Prevention” (AMP) has been initiated in order to assess the ability of VRC01 bnAb (specific for the CD4 binding site) to decrease the risk of HIV acquisition in humans (HVTN 704; available online: https://ampstudy.org). In addition, new delivery systems aimed to improve the efficacy and feasibility of passive antibody prophylaxis are being investigated. For instance, nonviral vector-mediated antibody gene transfer to express bnAbs in vivo may bypass traditional passive immunization, which would require long-term repeated treatments. In a recent study, it was shown that administration of lipid-encapsulated nucleoside-modified mRNA encoding heavy and light chains of VRC01 bnAb to humanized mice resulted in high serum antibody concentrations and protection against HIV challenge [89].

Another relevant option in preventing HIV infection is the so-called pre-exposure prophylaxis (PrEP), a pharmacological approach based on the daily oral administration of HIV integrase inhibitors to people at risk. To provide sustained drug delivery and circumvent concerns related to adherence to this treatment, long-acting formulation of antiretroviral drugs have been developed and tested in clinical trials [90].

## 8. Financial Concerns

On 28 March 2018, the World Health Organization (WHO) convened (in Geneva, Switzerland) researchers, policy makers, and AIDS activists to discuss the factors—including cost and ease of delivery—that can determine whether an HIV vaccine can succeed and how to prioritize the studies in the field, given the limitations of resources [91]. Moreover, officials involved in regulatory agencies reported the difficulties of facing decisions when considering the cost and effort of delivering treatment to people at risk. Similar concerns are relevant to passive immunotherapy: antibodies are expensive and are given as intravenous infusions, and it is unclear how long the treatment must continue.

However, along with the concerns, the discussion of these important issues is crucial and may foster renewed efforts in the scientific community to focus their research and define the milestone to achieve in order to obtain a successful vaccine formulation against HIV.

## Figures and Tables

**Table 1:** HIV clinical trials, rationale design and outcome.

Study	Regimen	Participants	Aim	Outcome	References
VAX004 (United States, Netherlands)	*r*gp120 B/B	MSM, high-risk women	bnAbs	No prevention of HIV infection	[61,62]
VAX003 (Thailand)	*r*gp120 B/E	Drug users	bnAbs	No prevention of HIV infection	[63,64]
Step/HVTN502 (USA)	*r*Ad5 HIV-1 gag/pol/nef B	MSM, high-risk women	CD8+ T-cells	Increased infection risk	[67]
Phambili/HVTN503 (South Africa)	*r*Ad5 HIV-1 gag/pol/nef B	Heterosexual men, women	CD8+ T-cells	Increased infection risk	[68]
HVTN505	* DNA/*r*Ad5	MSM, transgender women	Ab and T-cells	No infection risk, no efficacy	[69]
RV144 (Thailand)	* ALVAC-HIV/AIDSVAX B/E gp120 in alum	High risk men and women	Ab and T-cells	31.2% vaccine efficacy	[7]

*r*: recombinant; MSM: men who have sex with men; bnAbs: broadly neutralizing antibodies; *: prime-boost regimen.

**Table 2:** Current HIV-1 vaccine approaches.

Study/Strategy	Regimen	Host	Concept	Outcome	References
RV305	RV144 with additional boosts	Uninfected RV144 vaccinees	Boosting the immune response	Expansion of CD4bs-specific memory B-cells	[74]
HVTN097 (South Africa)	RV144	Uninfected men and women	Testing RV144 efficacy in South Africa	Env-specific CD4+ T-cells	[75]
HVTN100 (South Africa)	ALVAC-HIV C/gp120 in MF59	Uninfected men and women	Enhancing and sustaining the immunity	ongoing	[76]
Mosaic vaccine	Ad26 HIV-1 gag/pol/env	NHP	Increasing breadth and depth of specific immunity	Polyfunctional Ab and cellular immune responses	[77,78]
SOSIP	*r*HIV-1 Env trimers	Rabbits, NHP	bnAb	Autologous Tier-2 nAbs	[39,81,82]
HVTN 704 (passive immunotherapy)	*m*nAb	MSM	Protection against infection	ongoing	Available online: https://ampstudy.org

*r*: recombinant; *m*: monoclonal; MSM: men who have sex with men; nAbs: neutralizing antibodies; CD4bs: CD4-binding site; NHP: non-human primate.
